# Comparison of denosumab and zoledronic acid for the treatment of solid tumors and multiple myeloma with bone metastasis: a systematic review and meta-analysis based on randomized controlled trials

**DOI:** 10.1186/s13018-021-02554-8

**Published:** 2021-06-22

**Authors:** Lianghai Jiang, Xianghua Cui, Haoning Ma, Xiangsheng Tang

**Affiliations:** 1 Department of Spinal Surgery, Qingdao Municipal Hospital, Qingdao, 266000 Shandong China; 2 Department of Gastroenterology, Qingdao Municipal Hospital, Qingdao, 266000 Shandong China; 3 Department of Spinal Surgery, China-Japan Friendship Hospital, Beijing, 100029 China

**Keywords:** Denosumab, Zoledronic acid, Bone metastasis, Meta-analysis

## Abstract

**Objective:**

To compare the efficacy and safety between denosumab and zoledronic acid for advanced cancer with bone metastasis.

**Methods:**

MEDLINE, EMBASE, and the Cochrane library databases were searched for randomized controlled trials up to December 2020 that compared denosumab and zoledronic acid in the treatment of advanced cancer with bone metastasis. The following clinical outcomes were extracted for analysis: time to first skeletal-related event, time to first-and-subsequent skeletal-related events, overall survival, and disease progression. Safety outcomes including incidence of adverse events, serious adverse events, acute-phase reactions, renal toxicity, osteonecrosis of the jaw, and hypocalcemia were also extracted.

**Results:**

Four randomized controlled trials involving 7201 patients were included. The overall analysis showed that denosumab was superior to zoledronic acid in delaying time to first skeletal-related event (hazard ratio = 0.86; 95% confidence interval, 0.80–0.93; P < 0.01) and time to first-and-subsequent skeletal-related events (risk ratio 0.87; 95% confidence interval 0.81–0.93; P < 0.01). Denosumab was associated with lower incidence of renal toxicity (risk ratio 0.69; 95% confidence interval 0.54–0.87; P < 0.01) and acute phase reaction (risk ratio 0.47; 95% confidence interval 0.38–0.56; P < 0.01), but higher incidence of hypocalcemia (risk ratio 1.78; 95% confidence interval 1.33–2.38; P < 0.01) and osteonecrosis of the jaw (risk ratio 1.41; 95% confidence interval 1.01–1.95; P = 0.04). No significant differences were found in overall survival, time to disease progression, or incidence of adverse events and serious adverse events between denosumab and zoledronic acid.

**Conclusions:**

Compared with zoledronic acid, denosumab is associated with delayed first-and-subsequent skeletal-related events, lower incidence of renal toxicity, and acute phase reaction, but higher incidence of hypocalcemia and osteonecrosis of the jaw. Hence, denosumab seems to be a promising choice for advanced cancer with bone metastasis. Nonetheless, more randomized controlled trials are needed for further evaluation.

## Introduction

Metastasis to bone is one of the common complications associated with different types of advanced cancers, including solid tumors and multiple myeloma [1]. Patients with bone metastasis are at the risk of skeletal-related events (SREs).SREs include pathological fractures, requirement for radiation or surgery to bone to prevent or repair major structural damage, and spinal cord compression [2]. SREs represents a significant cause of morbidity including functional impairment and loss of mobility, which often lead to tremendous burden on quality of life and overall health [3–7].

As one of the osteoclast inhibitors, bisphosphonates have been widely used for advanced cancer patients with bone metastasis. In particular, zoledronic acid (ZA) is more effective than other bisphosphonate in delaying the first SREs [8, 9]. ZA has been the standard choice to prevent bone metastasis-related skeletal complications for almost a decade [10, 11]. However, side effects of ZA, including renal impairments and acute-phase reactions, limited its overall application [12–15]. As an alternative therapeutic option, denosumab has also been found to be effective in delaying SREs in advanced cancer. As a human monoclonal antibody, denosumab binds to receptor activator of nuclear factor kappa-B ligand (RANKL) [16–18], and has been shown non-inferior to ZA.

Several studies and meta-analysis [19] have been conducted to compare the efficacy and safety between denosumab and ZA. However, previous meta-analysis was performed based on several different types of advanced cancers without subgroup analysis. Specifically, solid tumors are different form multiple myeloma to a great extent. Besides, several good original manuscripts have been added. It is necessary to conduct a new review for the comparison of denosumab and ZA. Therefore, we performed a systematic review and meta-analysis to compare the efficacy and safety between denosumab and ZA for advanced cancer with bone metastasis.

## Materials and methods

### Inclusion criteria

Studies meeting the following criteria were included: (1) target population: patients with bone metastasis secondary to advanced solid tumors or multiple myeloma, (2) intervention: denosumab versus ZA, (3) methodological criteria: randomized controlled double-blind trials (RCTs), and (4) involved human patient populations. If the same study was published in different years or journals, then the most frequently cited report was included. Case reports and reviews were excluded from the analysis.

### Search strategy

Relevant studies were identified by searching the following databases: MEDLINE, EMBASE, Web of Science, and the Cochrane Collaboration Library up to December 2020. We used the search terms “denosumab,” “zoledronic acid,” “bone metastasis,” “cancer,” “tumor,” “neoplasm,” “multiple myeloma,” and “randomized controlled trial” with different combinations of the operators “AND,” “NOT,” and “OR.” References cited in selected studies were also checked to identify additional studies. Two reviewers (L.J. and X.C.) screened the studies independently.

### Data extraction

Two reviewers (L.J. and X.C.) extracted data from the eligible studies independently. Information from each study was collected in terms of the following contents: year of publication, author, study design, study time periods, and patient characteristics. The following clinical outcomes were extracted from included studies for comparison: time to first SRE (defined as the time in days from randomization date to the date of first occurrence of an on-study SRE [20]), time to first-and-subsequent SREs (defined as the time in days from randomization date to the date of a subsequent occurrence of an on-study SRE [20]), overall survival, and disease progression. Incidence of treatment-emergent adverse events (AEs) and serious AEs were extracted for comparison. All AEs were coded using Medical Dictionary for Regulatory Activities v12.0 system. In addition, safety outcomes including acute-phase reactions, renal toxicity, osteonecrosis of the jaw (ONJ), hypocalcemia, and new primary malignancy were also extracted for comparison.

### Quality assessment

Quality of the included RCTs were assessed by two reviewers (L.J. and H.M.) independently using the Cochrane assessment tool. Level of agreement between the two reviewers was recorded, and disagreements between them were resolved after the discussion with the third author (X.T.).

### Statistical analysis

Review Manager (Revman, version 5.3) was used for the data synthesis and analysis. Time-to-event data were pooled as hazard ratio (HR) and 95% confidence interval (CI), including time to first SRE, and time to first-and-subsequent SREs. Dichotomous data was pooled as risk ratio (RR) and 95% CI. Statistical heterogeneity was assessed using the χ^2^ test. P < 0.10 or I^2^ > 50% indicated significant heterogeneity. A fixed-effect model was used if low statistical heterogeneity was reported; otherwise, a random-effects model was used. P < 0.05 indicated statistically significant difference.

## Results

### Literature search

**Fig. 1:**
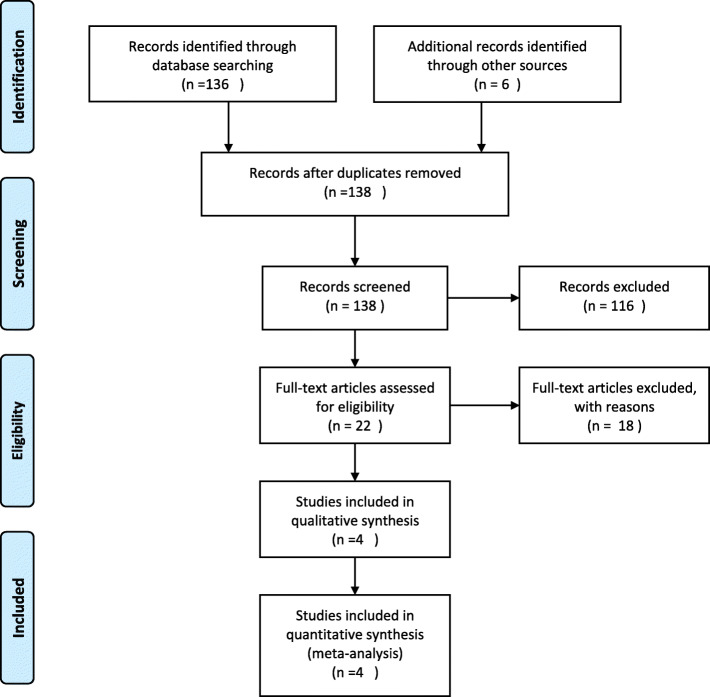
Flow of studies through review

We identified 138 articles that could potentially be included in the analysis after search of published literature. A total of 134 articles were excluded for not meeting inclusion criteria after reviewing title, abstract, or full text, resulting in four [20–23] randomized controlled trials. Detailed steps of the literature search are shown in Fig. 1.

### Study characteristics

Four RCTs [20–23] met the inclusion criteria, comparing the efficacy and safety of denosumab with ZA in advanced cancer with bone metastasis. Sample sizes of the four studies ranged from 1578 to 2033. Overall, 7201 patients were enrolled in these studies, including 3605 in the denosumab group and 3596 in the ZA group. Baseline characteristics of the four studies are provided in Table 1.

**Table 1:** Baseline characteristics of the studies included

Study	Cancer type	Interventions	Follow-up (mon)	N (denosumab/ZA)	Primary endpoint
Stopeck et al. (2010) [22]	Breast cancer	Denosumab 120 mg SC versus ZA 4 mg IV	33	1026/1020	Time to first SRE
Fizazi et al. (2011) [21]	Prostate cancer	Denosumab 120 mg SC versus ZA 4 mg IV	40.5	943/945	Time to first SRE
Henry et al. (2014) [23]	Various solid tumors	Denosumab 120 mg SC versus ZA 4 mg IV	34	800/797	Time to first SRE
Raje et al. (2018) [20]	Multiple myeloma	Denosumab 120 mg SC versus ZA 4 mg IV	50	859/859	Time to first SRE

### Quality assessment

**Fig. 2:**
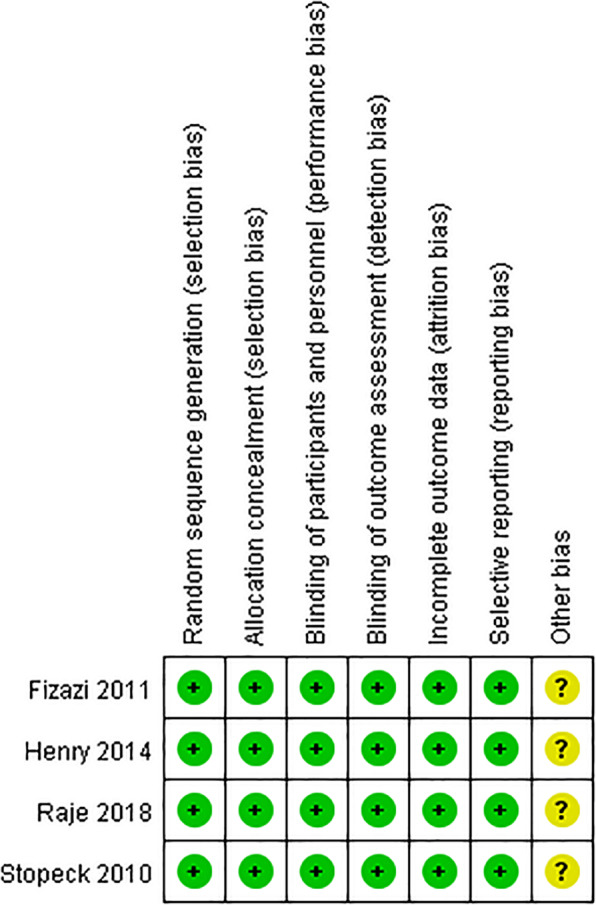
Quality assessment of the 4 randomized controlled trials included

All the included four RCTs were phase 3 study of denosumab versus zoledronic acid in patients with advanced cancer. Quality of the included studies was evaluated using the Cochrane assessment tool. The assessment of various items revealed a low risk of bias among the included four studies (Fig. 2).

### Assessing efficacy

**Fig. 3:**
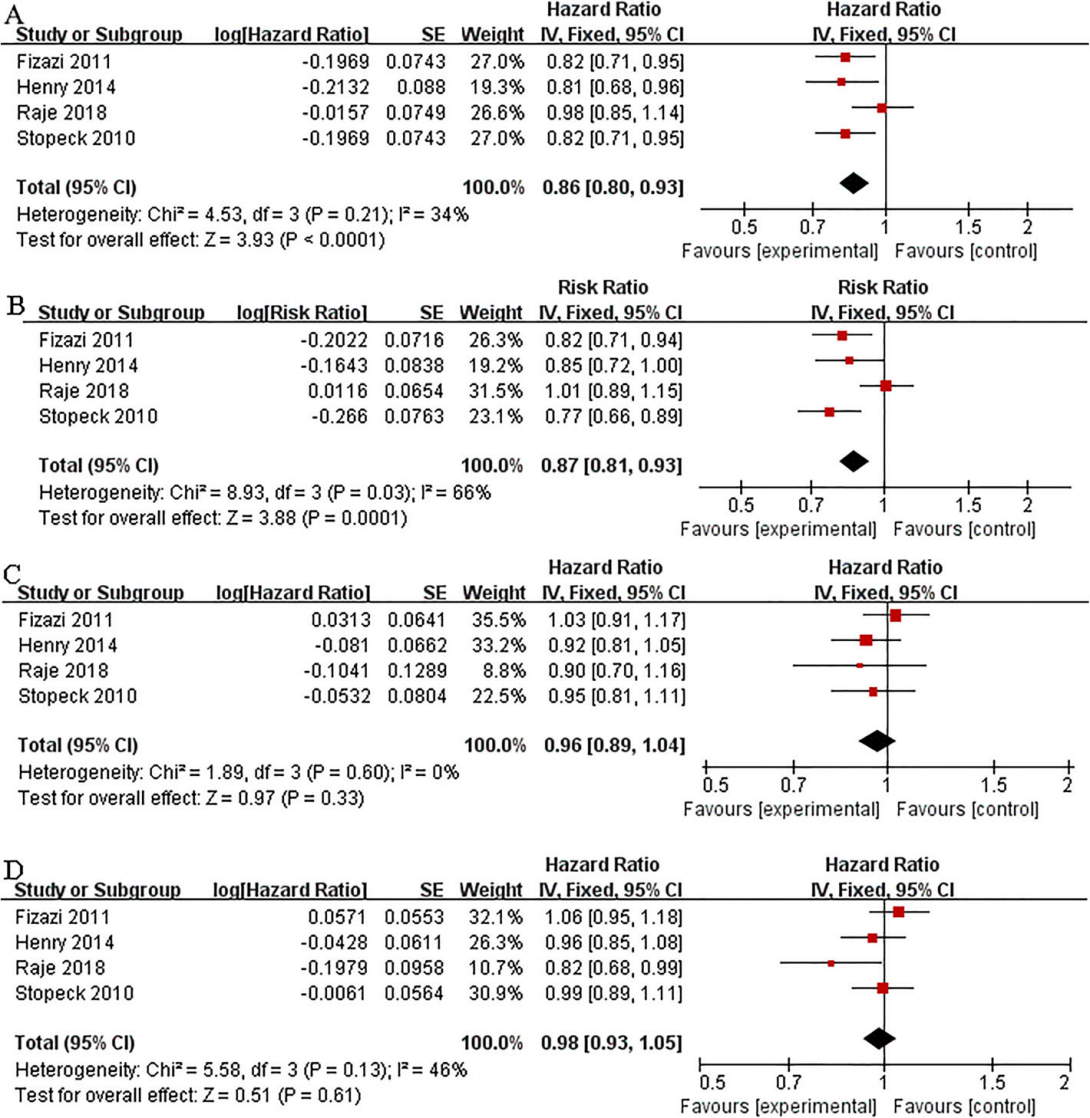
Overall analysis of denosumab versus ZA. Forest plots of time to first SRE (**A**), time to first-and-subsequent SREs (**B**), overall survival (**C**), and time to disease progression (**D**)

All the four studies presented information about time to first and first-and-subsequent SREs, overall survival improvement, and time to disease progression. In the study by Henry et al. [23], the median time to first on-study SRE was 21.4 and 15.4 months in patients receiving denosumab (n = 800) and ZA (n = 797) respectively (hazard ratio (HR) 0.81; 95% confidence interval (CI) 0.68–0.96; P = 0.017). In the study by Fizazi et al. [21], the median time to first on-study SRE was 20.7 and 17.1 months in patients receiving denosumab (n = 950) and ZA (n = 951) respectively (HR 0.82; 95% CI 0.71-0.95; P = 0.0002). In the study by Raje et al. [20], the median time to first on-study SRE was 22.8 and 24.0 months in patients receiving denosumab (n = 859) and ZA (n = 859) respectively (HR 0.98; 95% CI 0.85-1.14; P = 0.010). The pooled result showed that denosumab was significantly superior to ZA in delaying time to first SRE (HR 0.86; 95% CI 0.80–0.93; P < 0.01), and time to first-and-subsequent SREs (risk ratio (RR) 0.87; 95% CI 0.81–0.93; P < 0.01). No significant differences were found between two groups in overall survival (HR 0.96; 95% CI 0.89–1.04; P = 0.33), or time to disease progression (HR 0.98; 95% CI 0.93–1.05; P = 0.61) (Fig. 3).

**Fig. 4:**
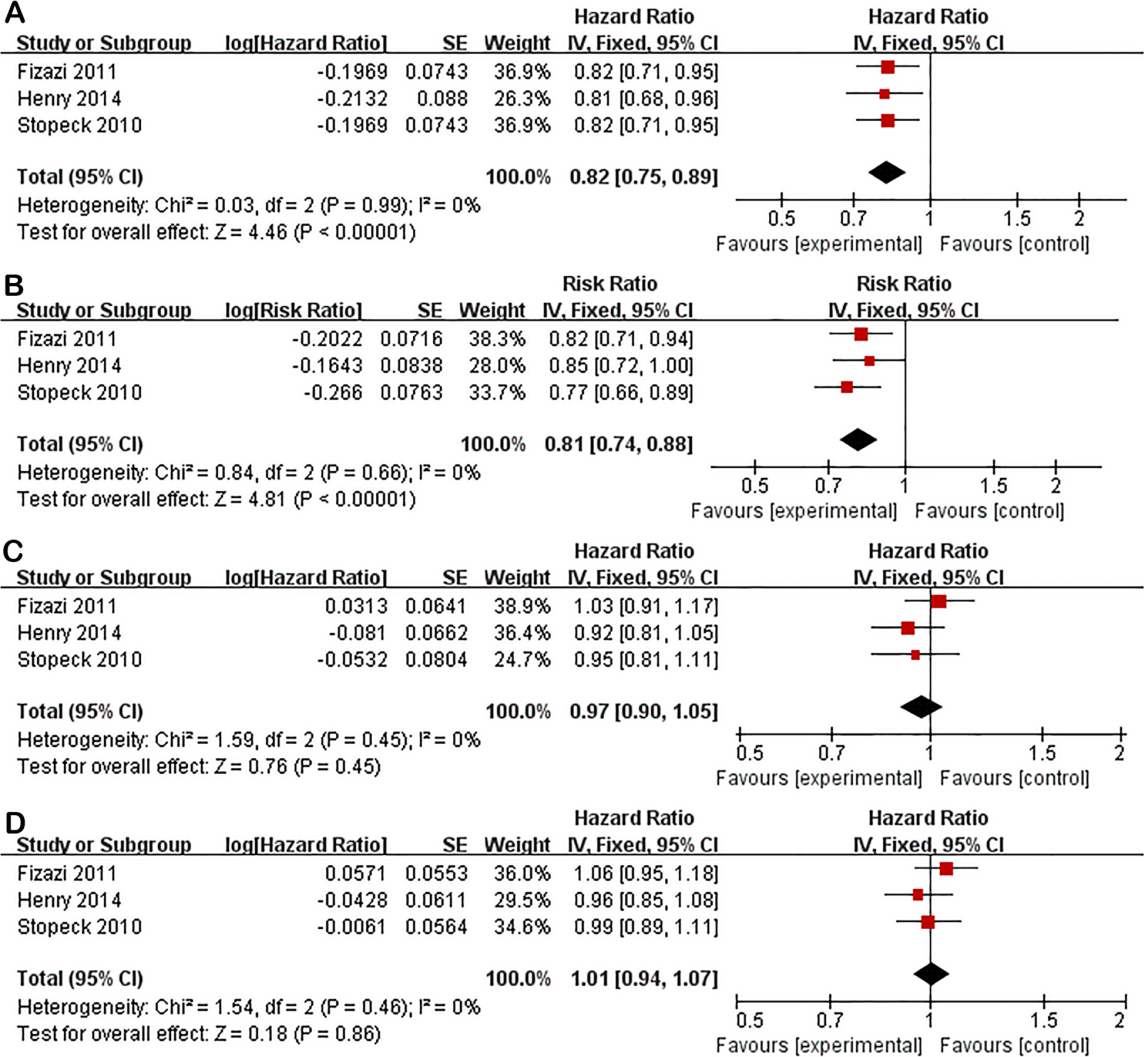
Subgroup analysis of denosumab versus ZA in solid tumors. Forest plots of time to first SRE (**A**), time to first-and-subsequent SREs (**B**), overall survival (**C**), and time to disease progression (**D**)

Excluding multiple myeloma, subgroup analysis was carried out between denosumab and ZA in solid tumors with bone metastasis. Pooled results showed that denosumab was superior to ZA in delaying time to first SRE (HR 0.82; 95% CI 0.75–0.89; P < 0.01) and time to first-and-subsequent SREs (RR 0.81; 95% CI 0.74–0.88; P < 0.01). There were no significant differences between two groups in overall survival (HR 0.97; 95% CI 0.90–1.05; P = 0.45), or time to disease progression (HR 1.01; 95% CI 0.94–1.07; P = 0.86) (Fig. 4).

### Assessing safety

**Fig. 5:**
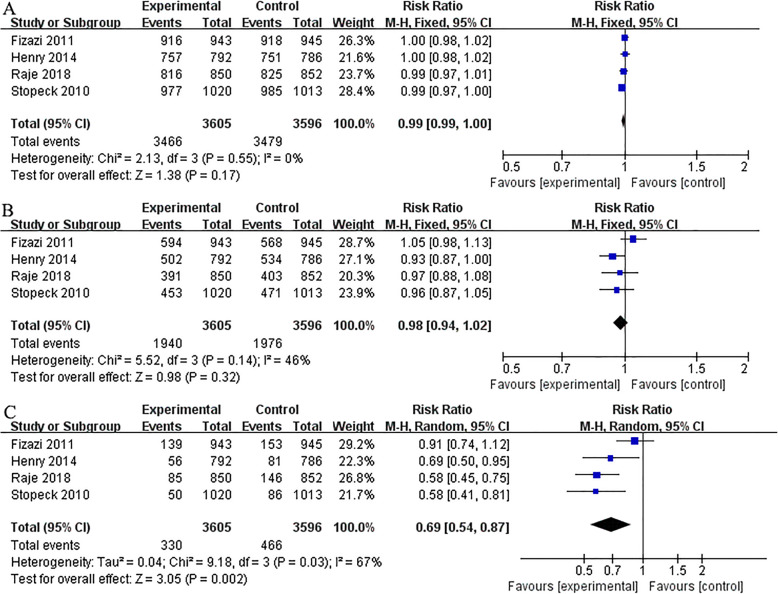
Overall analysis of denosumab versus ZA. Forest plots of incidence of AE (**A**), serious AEs (**B**), and renal toxicity (**C**)

**Fig. 6:**
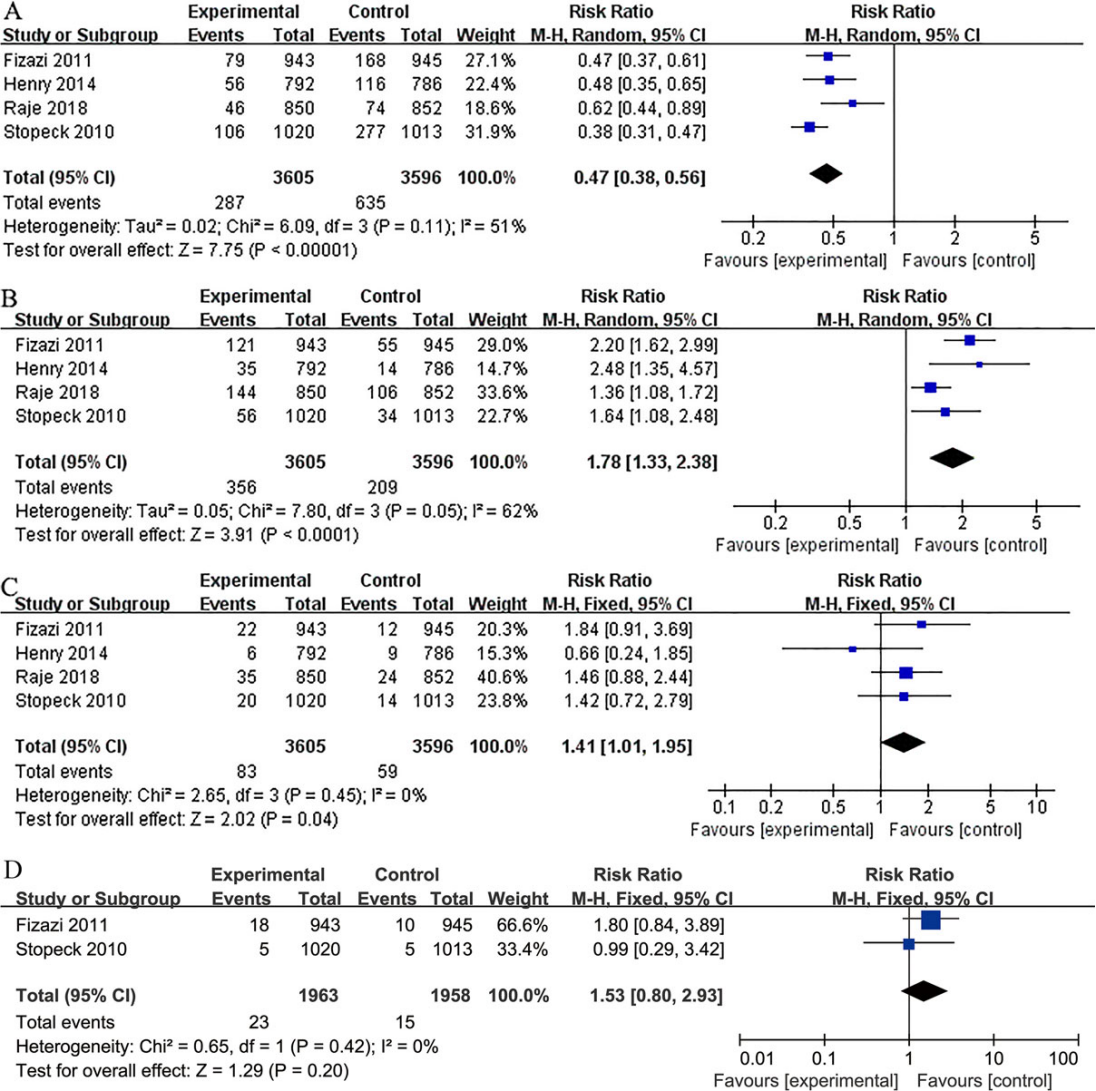
Overall analysis of denosumab versus ZA. Forest plots of incidence of acute phase reaction (**A**), hypocalcaemia (**B**), ONJ (**C**), and new primary malignancy (**D**)

All the included four studies presented information about overall AE, serious AEs, acute-phase reactions, renal toxicity, ONJ, and hypocalcemia. No significant differences were found in the incidence of AE (RR 0.99; 95% CI 0.99–1.00; P = 0.17), serious AEs (RR 0.98; 95% CI 0.94–1.02; P = 0.32) between denosumab and ZA. Pooled results showed that denosumab was associated with lower incidence of renal toxicity (RR 0.69; 95% CI 0.54–0.87; P < 0.01), and acute phase reaction (RR 0.47; 95% CI 0.38–0.56; P < 0.01). However, denosumab was also associated with higher incidence of hypocalcemia (RR 1.78; 95% CI 1.33–2.38; P < 0.01), and ONJ (RR 1.41; 95% CI 1.01–1.95; P = 0.04). Two studies [21, 22] presented information about incidence of new primary malignancy. No significant differences were found between denosumab and ZA (RR 1.53; 95% CI 0.80–2.93; P = 0.20) (Figs. 5 and 6).

**Fig. 7:**
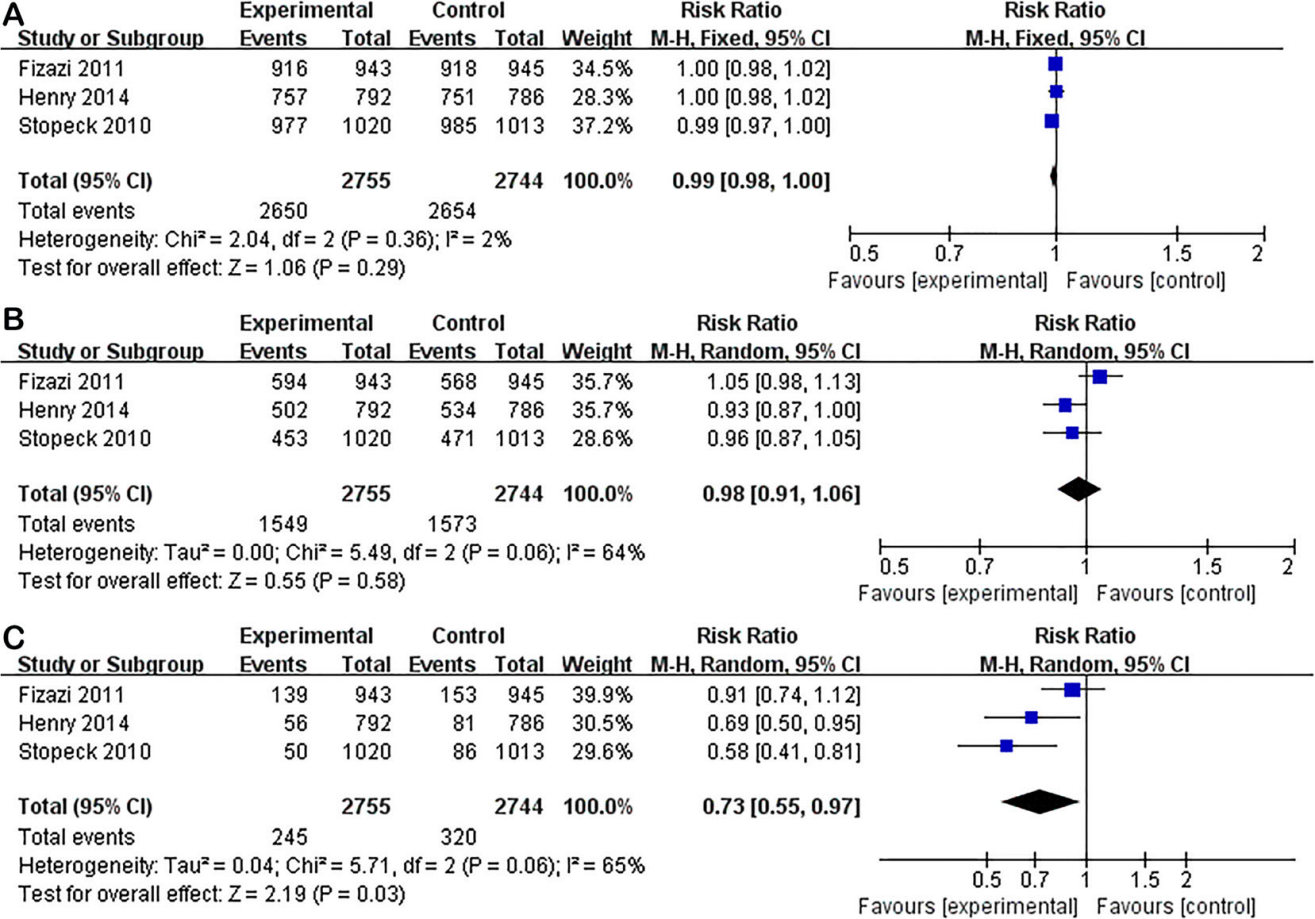
Subgroup analysis of denosumab versus ZA in solid tumors. Forest plots of incidence of AE (**A**), serious AEs (**B**), and renal toxicity (**C**)

**Fig. 8:**
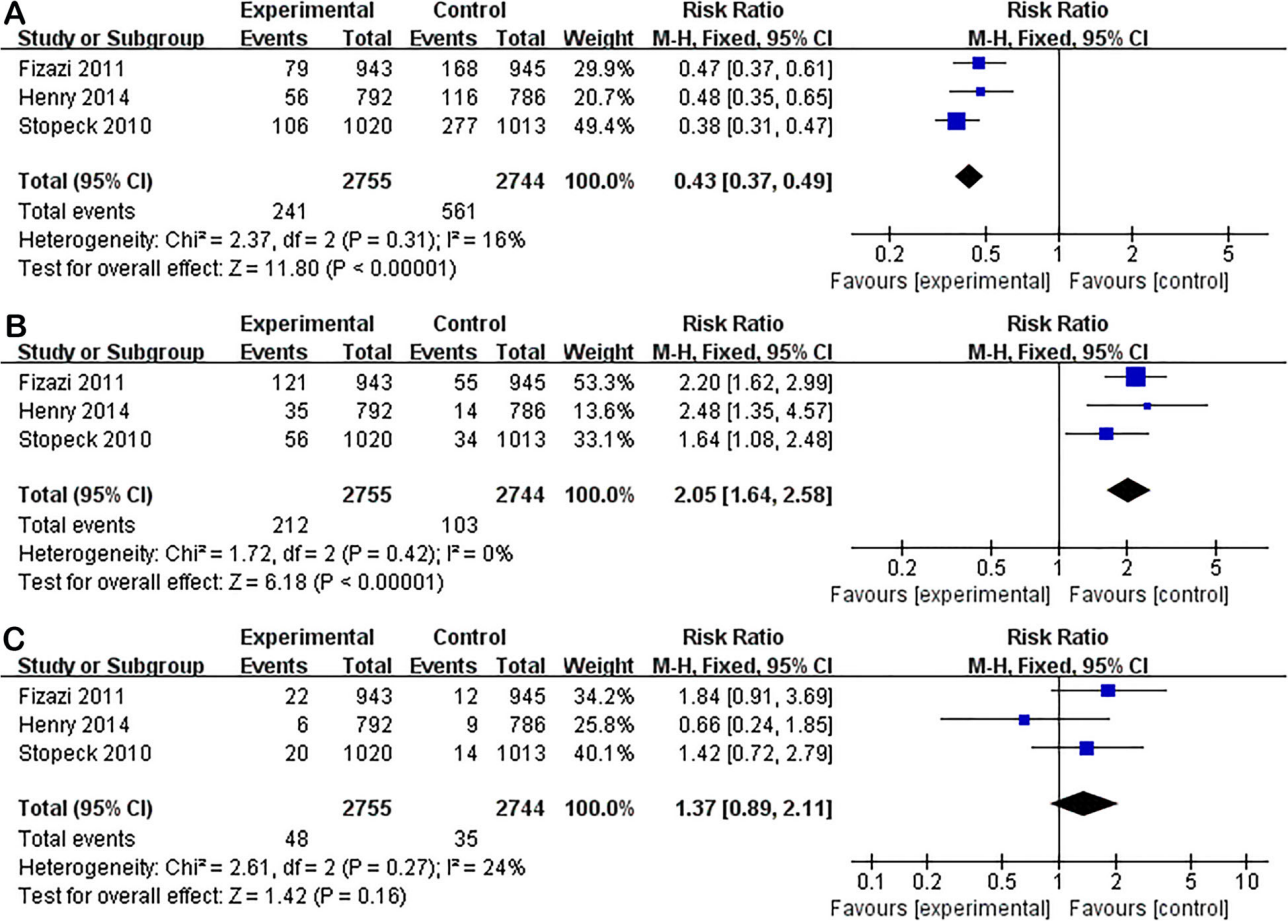
Subgroup analysis of denosumab versus ZA in solid tumors. Forest plots of incidence of acute phase reaction (**A**), hypocalcaemia (**B**), and ONJ (**C**)

Subgroup analysis between two groups in solid tumors with bone metastasis also showed no significant differences in AE (RR 0.99; 95% CI 0.98–1.00; P = 0.29) or serious AEs (RR 0.98; 95% CI 0.91–1.06; P = 0.58). Denosumab was related to lower incidence of renal toxicity (RR 0.73; 95% CI 0.55–0.97; P = 0.03) and acute phase reaction (RR 0.43; 95% CI 0.37–0.49; P < 0.01), and higher incidence of hypocalcemia (RR 2.05; 95% CI 1.64–2.58; P < 0.01). Unlike the overall analysis, subgroup analysis showed no significant differences in the incidence of ONJ (RR 1.37; 95% CI 0.89–2.11; P = 0.16) (Figs. 7 and 8).

## Discussion

For years, ZA has been regarded as the standard of care for advanced cancer with bone metastasis to prevent SREs [24, 25]. However, SREs continue to occur, albeit at a reduced rate. There are also several limitations and inconveniences for ZA use, including need for intravenous access, monitoring of renal function, need for dose adjustment in patients with renal impairment, and management of acute phase reactions. By contrast, denosumab does not have these limitations, which is given subcutaneously, and not associated with renal toxicity or acute phase reactions [26–29].

In this meta-analysis, four RCTs [20–23] of 7201 patients with high quality were included. All the included four RCTs were phase 3 study of denosumab versus zoledronic acid in patients with advanced cancer. With respect to time to first SRE and time to first-and-subsequent SREs, pooled results showed that denosumab was significantly superior to ZA in delaying time to first SRE and time to first-and-subsequent SREs. This finding was consistent with three RCTs [21–23] except the study by Raje et al. [20]. In the RCT by Raje et al. [20] with 1718 multiple myeloma patients, no significant differences were found between denosumab and ZA in time to first SRE or first-and-subsequent SREs. Subgroup analysis was consistent with the overall analysis.

With regard to overall survival and time to disease progression, both overall analysis and subgroup analysis showed no differences between denosumab and ZA. One thing to note is that Raje et al. [20] reported superiority of denosumab to ZA in delaying time to disease progression, which was different from the other three studies. As multiple myeloma is different from solid tumor, it is important to conduct subgroup analysis.

As to safety assessment in this meta-analysis, both overall analysis and subgroup analysis showed no differences between denosumab and ZA in overall AE or serious AEs. Pooled results showed that denosumab was associated with lower incidence of renal toxicity and acute-phase reactions. Subgroup analysis showed similar results. Pooled results also showed that denosumab was associated with higher incidence of hypocalcemia and ONJ. Interestingly, all the included four studies [20–23] showed no significant difference in the incidence of ONJ, which was inconsistent with the pooled result. And subgroup analysis based on solid tumor showed no difference in the incidence of ONJ. So, further analysis with more studies is needed for the comparison of incidence of ONJ. As reported, ONJ was usually related with previously reported risk factors, such as tooth extraction [30]. Probably due to its higher antiresorptive potency over ZA, denosumab was associated with higher incidence of hypocalcemia, which was consistent with all the included studies [20–23]. As to the incidence of new primary malignancy, Chen and Pu [31] reported denosumab was associated with higher incidence, which was inconsistent with the three studies included [21, 32, 33]. However, our meta-analysis showed no significant difference between denosumab and ZA, which was consistent with the two studies included [21, 22]. More studies are needed for further analysis.

Based on this systematic review and meta-analysis, denosumab is associated with delayed first and subsequent SREs, lower incidence of renal toxicity, and acute-phase reactions, but higher incidence of hypocalcemia and ONJ. Small number of included studies is the main limitation of this meta-analysis. Further analysis with more studies is needed for the comparison of denosumab and ZA for advanced cancer with bone metastasis.

## Data Availability

The data and materials contributing to this article may be made available upon request by sending an e-mail to the first author.

## Abbreviations

SREs: Skeletal-related events
ZA: Zoledronic acid
AEs: Adverse events
ONJ: Osteonecrosis of the jaw
HR: Hazard ratio
CI: Confidence interval
RR: Risk ratio
